# Primary and Secondary Prophylaxis of Gastrointestinal Bleeding in Children with Portal Hypertension: A Multicenter National Study by SIGENP

**DOI:** 10.3390/children12070940

**Published:** 2025-07-17

**Authors:** Naire Sansotta, Paola De Angelis, Daniele Alberti, Fabiola Di Dato, Serena Arrigo, Matteo Bramuzzo, Benedetta Calcaterra, Mara Cananzi, Maurizio Cheli, Andrea Chiaro, Francesco Cirillo, Mara Colusso, Grazia Di Leo, Simona Faraci, Paola Gaio, Giuseppe Indolfi, Silvia Iuliano, Daniela Liccardo, Antonio Marseglia, Matteo Motta, Federica Nuti, Filippo Parolini, Sara Renzo, Francesca Sbravati, Marco Sciveres, Claudia Mandato, Angelo Di Giorgio

**Affiliations:** 1 Paediatric Hepatology Gastroenterology and Transplantation, Hospital Papa Giovanni XXIII, 24127 Bergamo, Italy; 2 Gastroenterology and Nutrition Unit, Bambino Gesù Children’s Hospital IRCCS, 00165 Rome, Italy; paola.deangelis@opbg.net; 3 Department of Pediatric Surgery, ASST Spedali Civili, 25100 Brescia, Italy; daniele.alberti@unibs.it (D.A.); filippo.parolini@asst-spedalicivili.it (F.P.); 4 Department of Translational Medical Science, University of Naples Federico II, 80121 Naples, Italy; fabiola.didato@unina.it; 5 Pediatric Gastroenterology and Endoscopic Unit, IRCCS Istituto Giannina Gaslini, 16121 Genoa, Italy; serenaarrigo@gaslini.org (S.A.); andreachiaro@tiscali.it (A.C.); 6 Institute for Maternal and Child Health IRCCS “Burlo Garofolo”, 34121 Trieste, Italy; matteo.bramuzzo@burlo.trieste.it (M.B.); grazia.dileo@burlo.trieste.it (G.D.L.); 7 Department of Pediatrics, University of Milano—Bicocca, 20100 Milan, Italy; b.calcaterraborri@campus.unimib.it; 8 University Hospital of Padova, 35121 Padua, Italy; mara.cananzi@aopd.veneto.it (M.C.); paola.gaio@aopd.veneto.it (P.G.); 9 Paediatric Surgery, Hospital Papa Giovanni XXIII Bergamo, 24127 Bergamo, Italy; mcheli@asst-pg23.it (M.C.); mcolusso@asst-pg23.it (M.C.); 10 Santobono-Pausilipon Children’s Hospital, 80121 Naples, Italy; f.cirillo@santobonopausilipon.it; 11 Gastroenterology and Nutrition, Bambino Gesù Children’s Hospital IRCCS, 00163 Rome, Italy; simona.faraci@opbg.net; 12 Meyer Children’s Hospital IRCCS, 50139 Florence, Italy; giuseppe.indolfi@meyer.it; 13 Gastroenterology and Endoscopy Unit, University Hospital of Parma, 43126 Parma, Italy; siuliano@ao.pr.it; 14 Unit of Hepatology and Liver Transplant, Division of Metabolic Diseases and Hepatology, Bambino Gesù Children’s Hospital IRCCS, 00163 Rome, Italy; daniela.liccardo@opbg.net; 15 Fondazione IRCCS Casa Sollievo della Sofferenza, Division of Pediatrics, 71013 San Giovanni Rotondo, Italy; a.marseglia@operapadrepio.it; 16 University Hospital Parma, 43126 Parma, Italy; motta.matteo@asst-pg23.it; 17 Fondazione IRCCS Ca’ Granda Ospedale Maggiore Policlinico, 20129 Milan, Italy; federica.nuti@policlinico.mi.it; 18 Gastroenterology and Nutrition Unit, Meyer Children’s Hospital IRCCS, 50139 Florence, Italy; sara.renzo@meyer.it; 19 Pediatric Gastroenterology Unit, Maggiore Hospital, 40133 Bologna, Italy; francesca.sbravati@ausl.bologna.it; 20 ISMETT, University of Pittsburgh Medical Center Italy, 90133 Palermo, Italy; msciveres@ismett.edu; 21 Department of Medicine, Surgery and Dentistry “Scuola Medica Salernitana”, Pediatrics Section, University of Salerno, 84131 Baronissi, Italy; cmandato@unisa.it; 22 Department of Medicine, Pediatrics Section, University of Udine, 33100 Urdine, Italy; angelo.digiorgio@uniud.it

**Keywords:** portal hypertension, children, gastrointestinal bleeding

## Abstract

*Background/Objectives*: Portal hypertension (PH) is a common complication in children with chronic liver diseases. Primary and secondary prophylaxis of variceal bleeding in these patients remains controversial. Our study aims to evaluate the management of gastrointestinal (GI) varices in children with PH in Italy. *Methods:* A questionnaire was sent to 21 major pediatric hepatology centers. It included 34 questions referring to the medical, endoscopic, radiological, and surgical management of GI varices. *Results*: Out of 21 centers, 16 returned a completed questionnaire (survey response rate 76%) with a high level of completeness. A total of 1206 children with PH were under follow-up. Splenomegaly associated with hypersplenism was the main indication for endoscopic surveillance in all centers (100%). Primary prophylaxis was performed with endoscopy plus non-selective beta-blockers (NSBBs) in 50%, endoscopy alone in 38%, and NSBBs alone in 12%. All centers managed acute variceal bleeding with endoscopy within 24 h, acid suppression, and octreotide infusion. Secondary prophylaxis of variceal bleeding was conducted using endoscopy (100%) and NSBBs (87%). Transjugular intrahepatic portosystemic shunt (TIPS) was considered a good option when endoscopic treatment failed in 94% of centers. *Conclusions*: In Italy, there is broad consensus among centers regarding the management of gastrointestinal varices in children with portal hypertension. All participating centers endorsed the use of endoscopic screening for children presenting with clinical signs of portal hypertension. Nonetheless, further research is essential to establish evidence-based guidelines and to improve overall quality of care.

## 1. Introduction

Portal hypertension (PH) is a common complication in children with chronic liver diseases, including cholestatic disorders, thrombosis of the portal vein, porto-sinusoidal vascular disease, and autoimmune liver disease. In some cases, children with liver fibrosis and PH may present acutely and require liver transplantation. Therefore, early diagnosis and appropriate management of PH complications are crucial to improve long-term outcomes with the native liver [1,2,3,4,5,6]. In pediatrics, the diagnosis of PH relies on finding evidence of splenomegaly and the formation of portosystemic collaterals [7]. One of the most serious complications of PH is gastroesophageal (GE) variceal bleeding, which represents a major source of morbidity and life-threatening events requiring prompt medical intervention [8,9,10]. While non-invasive tests can help identify children at risk of bleeding, endoscopy remains the gold standard for diagnosing and managing gastrointestinal bleeding in pediatric patients [8,9,11,12,13,14,15]. The pediatric literature on this topic is limited, and no universally accepted guidelines exist regarding indications and timing for primary and secondary prophylaxis of variceal bleeding [16]. The Baveno consensus conference, a prominent forum for PH management in adults, has recently begun incorporating pediatric expertise to formulate recommendations specific to children [8,17,18,19]. In clinical practice, many pediatric hepatologists recommend screening for varices, often as a prelude to primary prophylactic therapy, and most parents of children with PH prefer that their child undergo endoscopic evaluation to better understand the bleeding risk, despite the absence of strong evidence from the published literature [7,8,9,11]. Due to the lack of comprehensive studies on endoscopic screening, variceal treatment, and non-selective beta-blocker (NSBB) therapy, management strategies differ significantly worldwide, influenced by local resources and institutional expertise [7,8,9,11]. In light of these challenges, the Liver Disease and Endoscopy Working Groups of the Italian Society for Pediatric Gastroenterology, Hepatology and Nutrition (SIGENP) conducted a national survey to explore current strategies used in Italy to prevent and manage gastrointestinal bleeding in children with PH.

## 2. Materials and Methods

This cross-sectional, multicenter, web-based survey was conducted between October 2023 and November 2023. The survey concept was developed in April 2023 during the Liver and Endoscopy working group meeting of the Italian Society of Pediatric Gastroenterology, Hepatology and Nutrition (SIGENP) with the aim of collecting retrospective data on children with chronic liver disease and PH managed over the past 15 years. Following discussion with working group members and representatives from leading pediatric gastroenterology centers in Italy, the authors identified the main controversies surrounding the diagnosis and management of PH, which were subsequently included in the questionnaire. The final version of the questionnaire was sent electronically to all SIGENP members. To maximize participation and ensure that the survey was representative of the major centers involved in managing PH across Italy, reminder emails were sent every two weeks.

The survey did not require disclosure of the responder’s identity, but only the name of the hospital and the city. The survey was distributed to 21 major pediatric gastroenterology and hepatology centers in Italy, in line with standard survey distribution practices. The study protocol conforms to the ethical guidelines of the 1975 Declaration of Helsinki (6th revision, 2008).

The major pediatric gastroenterology and hepatology centers included in the survey were selected based on the following predefined criteria to ensure their relevance and expertise in the field:

1. Institutional profile: University Hospital, Research Institute of High Specialization (IRCCS), tertiary-level referral center, or pediatric liver transplant center.

2. SIGENP affiliation: Each participating center had at least one SIGENP member affiliated with the working group in pediatric endoscopy, hepatology, or gastroenterology, thereby ensuring recognized experience and commitment to pediatric liver disease management.

The questionnaire consisted of 34 questions divided into four sections: (i) indications to screen for gastroesophageal varices, (ii) primary prophylaxis of variceal bleeding, (iii) management of acute variceal bleeding and secondary prophylaxis, and (iv) role of radiology and surgery for the management of gastrointestinal varices.

The rationale was divided into four specific sections with the aim of including the typical clinical trajectory of children with PH, thereby ensuring both logical flow and clinical relevance.

The questions within each section were formulated collaboratively by a panel of pediatric hepatologists and endoscopists, taking into account the most clinically relevant issues and areas of practice variability. We aimed to ensure clarity and comprehensiveness by using precise and unambiguous language, avoiding overly technical jargon where possible, and by including multiple-choice options that reflect real-world practice variations.

To answer the questionnaire, we used the following definitions to classify oesophageal varices. Grade 0: no oesophageal varices; grade 1: small, extending just above the mucosal level; grade 2: tortuous oesophageal varices but occupying less than one-third of the distal oesophagus radius; grade 3: large and tortuous oesophageal varices covering more than one-third of the distal oesophagus radius. Furthermore, gastroesophageal varices were classified into type 1 (GOV1) and type 2 (GOV2) [20].

Primary prophylaxis was defined as the treatment to prevent the first variceal bleeding, and secondary prophylaxis as the treatment to prevent recurrent variceal bleeding [8]. The overall survey response rate was defined as the ratio between the number of centers that responded to the number of Italian Pediatric Liver Centers that received the questionnaire.

## 3. Results

Of the 21 centers invited to participate, 16 returned fully completed questionnaires, yielding a response rate of 76% with a high degree of data completeness and representation of the north, center, and south of Italy. These centers collectively reported follow-up of 1206 children with portal hypertension (aged 1–18 years). The most common underlying diagnoses were biliary atresia (35%), portal vein thrombosis (33%), cystic fibrosis (7%), hepatic fibrosis (5%), and other conditions (20%).

**Screening for varices**: All centers (100%) screened for upper varices, 47% (7/15 centers) regardless of the underlying liver disease (cirrhotic or non-cirrhotic). Splenomegaly associated with hypersplenism (defined as platelet count <150,000 mm^3^) was the main indication for endoscopic surveillance in all centers (100%).

If no varices were found, 75% (12/16) of centers agreed to perform endoscopic surveillance at 12 (5/16:31%) or 24 (7/16:44%) months, while 25% of centers (4/16) repeated it only if PH worsened. Endoscopic surveillance to screen children who were listed for liver transplantation was performed in 14 out of 16 pediatric transplant centers (88%). Unlike in adults, non-invasive scoring systems, such as the platelet count/spleen length z-score ratio, platelet count alone, and the aspartate aminotransferase-to-platelet ratio index (APRI) [21,22] were not routinely used in pediatrics for screening children with PH.

**Primary prophylaxis:** All centers (100%) performed primary prophylaxis for high-risk varices, defined as any grade of varices with red signs or grade 3 varices. Both types of gastroesophageal varices (GOV1 and GOV2) were treated in 65% of centers.

Primary prophylaxis was performed as follows:

(a) Endoscopy and NSBBs in 8/16 centers (50%);
(b) Endoscopy alone in 6/16 (38%);
(c) NSBBs alone in 2/16 (12%).

**Figure 1:**
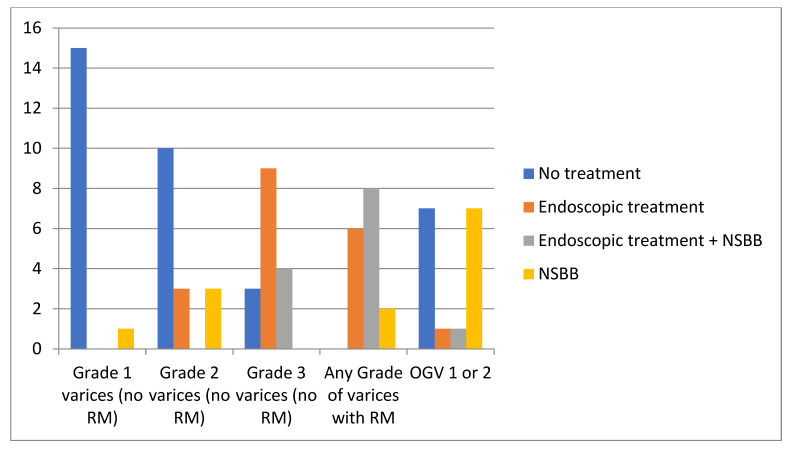
Primary prophylaxis of variceal bleeding based on endoscopic features of PH in 16 pediatric centers. NSBBs: Non-selective beta-blockers; RM: red marks; OGV: esophagogastric varices.

Children with grade 3 varices without red marks were treated mainly with endoscopy alone (9/16: 56%). Most centers did not treat grade 1 (94%) or grade 2 (63%) varices without red marks (Figure 1). Overall, NSBBs were used by 81% of centers, and propranolol was the drug of choice (100%), with dosing adjusted based on heart rate in 85% of centers (11/13); only 15% (2/13) administered a fixed dose. No major side effects were reported.

Variceal ligation was the standard endoscopic treatment in all centers (100%).

Remarkably, in children weighing <10 kgs, out of 16 centers, 10 (63%) used sclerotherapy as the standard treatment of choice due to technical limitations, such as the difficulty in inserting endoscopes capable of ligation in very small patients. The major complications related to endoscopic treatment (ligation and sclerotherapy) were pain and mild bleeding. No major adverse effects were reported.

**Acute bleeding and secondary prophylaxis:** All centers managed acute variceal bleeding with endoscopy within 24 h, acid suppression, and octreotide infusion (Table 1A).

Admission to the intensive care unit was recommended in 38% of centers (6/16), even for hemodynamically stable patients. Secondary prophylaxis of variceal bleeding was conducted using endoscopy (100%) and NSBBs (87%) (Table 1A).

When conservative management (endoscopy and NSBBs) failed to control variceal hemorrhage, more invasive procedures were considered, including TIPS in 94% of centers (15/16), surgical shunts in 63% (10/16), and liver transplantation in 50% (8/16) (Table 1B).

## 4. Discussion

This is an extensive survey-based study that focused on the management of pediatric PH among gastroenterologists and hepatologists in Italy. The high response rate and the good geographical distribution minimize the risk of non-response bias and ensure that the collected data reflect real-world practices across the majority of major pediatric hepatology centers in the country.

The clinical course and therapeutic response of bleeding from gastrointestinal varices in pediatric patients remain only partially understood. In this context, the present study seeks to evaluate the current strategies adopted across Italian healthcare centers with the aim of defining future prospective studies and providing recommendations.

Given the lack of standardized non-invasive tests in pediatric populations, our survey confirms the central role of endoscopic evaluation in clinical practice, consistent with findings reported in previous studies [9,11,23,24].

Remarkably, in a French study, more than 75% of centers declared to screen for GI varices in children with cirrhotic and non-cirrhotic PH [25]. These results demonstrated that, despite the absence of specific recommendations, there is tacit consensus on the need for screening children with PH through endoscopy.

All centers agreed on the indication for primary prophylaxis in cases of high-grade varices; however, the approaches varied significantly. Endoscopy remained the most commonly used intervention across the majority of centers, although 12% relied exclusively on NSBBs.

Similar findings were reported in the study by Jeanniard-Malet, where over 90% of centers implemented primary prophylaxis in children with high-risk varices, with 20% using NSBBs. In contrast, a Canadian study reported a lower overall rate of primary prophylaxis (58%), with 37% of centers employing NSBBs.

In a cohort of 1300 children, the authors reported that primary prophylaxis should be conducted in children with high-risk varices who do not require liver transplantation in the foreseeable future [9]. Variceal ligation was considered more effective and safe compared to sclerotherapy, as reported in the literature [11,23,25,26,27].

Therefore, our study follows the recommendation that sclerotherapy should be reserved for cases in which endoscopic band ligation (EBL) is not feasible, typically due to technical challenges in very small patients [28]. Conversely, while NSBBs are the preferred option for primary prophylaxis in adults with small varices at high risk of bleeding, evidence supporting their use in the pediatric population remains limited [29,30].

In Schneider’s study, the authors reported that NSBBs should be avoided due to there being limited evidence concerning dosage, safety, and efficacy [8]. Nevertheless, it has been reported by different authors that NSBBs are used predominantly in the setting of gastroesophageal varices [7]. In our study, more than half of the centers adopted primary prophylaxis with NSBBs (with or without endoscopy) with no side effects. However, we cannot speculate that NSBBs have a role in preventing gastrointestinal bleeding in our cohort.

In cases of acute bleeding, despite limited recommendations for use in the pediatric population, all centers routinely administer octreotide, reporting a very low incidence of adverse events. Although endoscopic treatment is effective in achieving variceal obliteration, it does not reduce portal pressure. Consequently, some centers consider radiological and/or surgical interventions following a bleeding episode, as supported by literature evidence^10^.

Of note, in cases of refractory gastrointestinal bleeding, TIPS placement is considered part of the armamentarium for the management of children with PH, supported by studies evaluating its feasibility and efficacy in children with both cirrhotic and non-cirrhotic PH [31,32,33,34].

Although, to our knowledge, the present study represents the most extensive data collection on current national practice concerning the diagnosis and management of children with PH, we have to acknowledge some limitations: the survey was designed to be rapid and easy to complete to involve a large number of centers, although the collected data lacked in precision; therefore, it does not guarantee the reliability of the results. Moreover, despite significant adherence, we cannot be sure that all of the centers that care for patients with PH responded to the questionnaire. However, the aforementioned wide geographical distribution and the high response rate are likely to be a reliable national representation.

## 5. Conclusions

This study aims to address current gaps in the management of pediatric portal hypertension by providing a comprehensive overview of real-world practices across expert centers. By capturing data on surveillance, prophylaxis, and treatment strategies, the survey highlights both areas of consensus and significant variability. These insights help identify where clinical uncertainty persists and where standardized, evidence-based guidelines are most needed.

We found that there is a high level of agreement among Italian centers in the management of gastrointestinal bleeding in pediatric portal hypertension, underscoring the effectiveness of the collaborative network led by the Liver Disease and Endoscopy Working Groups of SIGENP.

Endoscopic screening for varices is routinely performed in children presenting with splenomegaly and hypersplenism, and upper endoscopy represents a cornerstone in the management of children with PH. Primary prophylaxis commonly includes a combination of endoscopic interventions and NSBBs. Although few data are available about NSBBs and PH in children, we found a high degree of confidence in using them. A high grade of concordance was found in the acute bleeding setting. When conservative approaches prove ineffective, TIPS is regarded as a viable therapeutic option.

These findings underscore the urgent need for standardized, evidence-based guidelines for the management of children with portal hypertension. Future research should focus on developing a validated risk stratification model, including a diagnostic algorithm, and evaluating the efficacy and safety of non-selective beta-blockers in this specific patient population.

## Figures and Tables

**Table 1:** Management of children with gastrointestinal varices. (**A**) Treatment options for children with acute variceal bleeding; (**B**) Secondary prophylaxis after the first episode or recurrent variceal bleeding in children with portal hypertension.

(**A**)	
Acid suppression	100%
Octreotide	94%
Occlusion tube (e.g., Sengstaken–Blakemore)	38%
Intravenous antibiotics	63%
Endoscopy	100%
(**B**)	
*Treatment options after the first episode of variceal hemorrhage*	
NSBBs	87%
Endoscopic treatment (sclerotherapy or band ligation) *When Endoscopy and NSBB fail to control variceal hemorrhage*	100%
TIPS	94%
Surgical Shunt	63%
Transplantation	50%

NSBBs: non-selective beta-blockers; TIPS: transjugular intrahepatic portosystemic shunt.

## Data Availability

The original contributions presented in this study are included in the article. Further inquiries can be directed to the corresponding author.

## Abbreviations

The following abbreviations are used in this manuscript:

GI	Gastrointestinal
NSBBs	Non-selective beta-blockers
OGV	Esophagogastric varices
PH	Portal hypertension
RM	Red marks
TIPS	Transjugular Intrahepatic Portosystemic Shunt
